# A Novel Frizzled-Based Screening Tool Identifies Genetic Modifiers of Planar Cell Polarity in *Drosophila* Wings

**DOI:** 10.1534/g3.116.035535

**Published:** 2016-10-11

**Authors:** Jose Maria Carvajal-Gonzalez, Sonia Mulero-Navarro, Michael Smith, Marek Mlodzik

**Affiliations:** * Departamento de Bioquímica, Biología Molecular y Genética, Facultad de Ciencias, Universidad de Extremadura, 06071 Badajoz, Spain; † Department of Developmental and Regenerative Biology, Icahn School of Medicine at Mount Sinai, New York 10029; ‡ Graduate School of Biomedical Sciences, Icahn School of Medicine at Mount Sinai, New York 10029

**Keywords:** planar cell polarity, *Drosophila*, *frizzled*, *krasavietz*, *short*-*stop*

## Abstract

Most mutant alleles in the Fz-PCP pathway genes were discovered in classic *Drosophila* screens looking for recessive loss-of-function (LOF) mutations. Nonetheless, although Fz-PCP signaling is sensitive to increased doses of PCP gene products, not many screens have been performed in the wing under genetically engineered Fz overexpression conditions, mostly because the Fz phenotypes were strong and/or not easy to score and quantify. Here, we present a screen based on an unexpected mild Frizzled gain-of-function (GOF) phenotype. The leakiness of a chimeric Frizzled protein designed to be accumulated in the endoplasmic reticulum (ER) generated a reproducible Frizzled GOF phenotype in *Drosophila* wings. Using this genotype, we first screened a genome-wide collection of large deficiencies and found 16 strongly interacting genomic regions. Next, we narrowed down seven of those regions to finally test 116 candidate genes. We were, thus, able to identify eight new loci with a potential function in the PCP context. We further analyzed and confirmed *krasavietz* and its interactor *short-stop* as new genes acting during planar cell polarity establishment with a function related to actin and microtubule dynamics.

Planar cell polarity (PCP) is a highly conserved pathway that controls the orientation of single cells within a plane of an epithelium or tissue in general (Bayly and Axelrod 2011; Carvajal-Gonzalez and Mlodzik 2014; Goodrich and Strutt 2011; Seifert and Mlodzik 2007; Singh and Mlodzik 2012; Wang and Nathans 2007; Yang and Mlodzik 2015). In vertebrates, PCP signaling is essential for many developmental processes in epithelial organs, including for example the arrangement of hair follicles in the skin (Devenport and Fuchs 2008; Guo *et al.* 2004), the stereocilia in the inner ear (Montcouquiol *et al.* 2003), and cilia in airway epithelial cells (Vladar *et al.* 2012), but is also required in mesenchymal cell processes including chondrocyte orientation required for limb growth (Gao *et al.* 2011) or during gastrulation/neurulation processes (Solnica-Krezel and Sepich 2012), among other functions.

PCP was initially discovered in insects and first studied in detail in *Drosophila* wings, where each epithelial cell produces a single actin-based hair pointing distally following the polarized distribution of PCP proteins at the plasma membrane (Wong and Adler 1993). Subsequently, PCP was studied in other *Drosophila* tissues like the thorax, compound eye, or abdomen (Adler 2002; Klein and Mlodzik 2005; Lawrence and Casal 2013; Seifert and Mlodzik 2007; Strutt 2003). In the eye and thorax, PCP is reflected in the orientation of multi-cellular sensory units, the arrangement of photoreceptors in ommatidia in the eye, and sensory bristles on the thorax (Adler and Taylor 2001; Jenny 2010). In wings, when PCP signaling is perturbed, the actin-based hairs of each cell can point in semirandom directions and/or multiple actin hairs protrude from a single cell producing the so-called multiple wing hairs (mwh) (Wong and Adler 1993) or “multiple cellular hair” cell (mch) phenotype.

The conserved Frizzled-PCP signaling core pathway contains the plasma membrane proteins Frizzled (Fz), Strabismus/Van Gogh (Stbm/Vang, a four-pass TM protein, called Vangl in vertebrates), and Flamingo (Fmi, a.k.a. Starry night/Stan, an atypical cadherin, called Celsr in vertebrates), and the associated cytoplasmic Prickle (Pk), Dishevelled (Dsh, Dvl in mammals), and Diego (Dgo, Inversin/Diversin in vertebrates) (Adler 2002; Bayly and Axelrod 2011; Klein and Mlodzik 2005; Seifert and Mlodzik 2007; Strutt 2003; Wang and Nathans 2007). In addition to the Fz-PCP core group, a parallel and sometimes redundant pathway also acts on PCP signaling: the Fat (Ft)-Dachsous (Ds) PCP signaling pathway centered on the protocadherins Ft and Ds (Casal *et al.* 2006; Lawrence *et al.* 2007). In addition to the core PCP members, several PCP effector (PPE) genes have been identified. PPE mutant wings generally initiate two or more independent actin hairs at the apical membrane, causing the mch phenotype. Examples of PPE genes are *inturned* (*in*), *fuzzy* (*fy*), the anti-formin *multiple wing hairs* (*mwh*), *combover*, or *fritz* (frtz) among others (Adler 2012; Adler *et al.* 1994; Fagan *et al.* 2014; Lee and Adler 2002; Wang *et al.* 2014; Yan *et al.* 2008; Yun *et al.* 1999).

In recent years, our group has made an effort to identify new effectors and regulators of Fz-PCP signaling using genome-wide screens. These efforts have contributed to our knowledge of the biology of the PCP pathway, including the transport and signaling of core Fz-PCP proteins and identification of novel effectors and regulators of the core PCP complexes. For example, a genetic modifier screen using Dgo- and Pk-associated genotypes, a combination of large genomic deletions, and UAS-RNAi lines identified, among others, CK1-γ (*gilgamesh*, *Gish*) (Gault *et al.* 2012; Weber *et al.* 2012) and a PI4KIIIβ gene (*Four wheel drive*), which we used to unravel the requirement for Arf-1 and AP-1 in PCP establishment (Carvajal-Gonzalez *et al.* 2015; Weber *et al.* 2012). In addition, a forward EMS screen identified *furrowed* (*fw*), a *Drosophila* Selectin ortholog, acting as a cell adhesion molecule that mediates Fz membrane stability and hence the interaction between Fz and Vang/Stbm (Chin and Mlodzik 2013).

In the present study, we took advantage of a highly reproducible, yet mild *frizzled* overexpression system, thus suited for a modifier screen, to isolate several potential new regulators or effectors of PCP employing a genetic modifier screen. Out of the 279 large deficiencies initially screened, 19 deficiencies showed interactions with our Fz-mediated PCP phenotype. Within 7 of those 19 positive deficiencies, we identified eight specific genes as potential PCP factors and one, the elongation initiator factor *krasaviezt* (*kra*), was further confirmed as a PCP interactor. Among the new candidates, we found two mitochondrial related proteins (mRpL12 and mRpL35), a nucleoporin (CG14712), two D transcription factors [(*Atu*) and dichaete (D)], and Sem1.

## Materials and Methods

### Fly stocks

Flies were raised on standard medium and maintained at 25°, unless otherwise indicated. The GAL4/UAS system (Brand and Perrimon 1993) was used for gene expression and RNAi studies. The Gal4 expression drivers were as follow: *en-GAL4*, *nub-GAL4*, *dpp-GAL4*, *and heat-shock-GAL4*.

In addition, the following lines were used: *shot[3]*(gift from Katja Röper), *kakp^1^* (gift from Katja Röper), and *kra^1^*(gift from Seungbok Lee); *fz* RNAi (VDRC v43075), Fz1, and Fz121 transgenes [described in Wu *et al.* (2004)], *dsh^V26^*, *dgo^380^*, *pk-sple^6^*, *fz^p21^*, *aPKC*^-^, and *scrib* mutants (Bloomington Stock Center); and Shot-L(C)-GFP and *Shot JF0297* (Bloomington Stock Center). Additional RNAi lines from VDRC are listed in Supplemental Material, Table S1, Table S2, and Table S3. DrosDel deficiencies were received from Szeged (distributed by FlyBase, Ryder *et al.* 2007); Exelixis deficiencies (distributed by FlyBase) were from Harvard/Exelixis (Parks *et al.* 2004).

### Immunostaining and histology

To analyze trichome orientation and number in adult flies, wings were removed, washed in PBT buffer (PBS and 0.1% Triton X-100), and mounted in 80% glycerol (diluted in PBS) on a slide. Adult wings were scored and imaged at room temperature on a microscope (Axioplan; Carl Zeiss). Images were acquired with a camera (Zeiss AxioCam Color type 412–312; Carl Zeiss) and AxioCam software.

For analysis of pupal wings, prepupae (white pupae) were collected and staged at 25° for 30–32 hr for pupal wings. Wings were dissected and fixed in PBS with 4% formaldehyde and 0.1% Triton X-100 for 45 min. Tissues were washed twice in PBT and incubated in PBT with 2% BSA for 30–45 min. Primary antibodies were incubated overnight at 4°. Samples were washed five times in PBT and incubated for 1 hr with fluorescent secondary antibodies diluted in PBT. Five additional washes in PBT were performed before mounting on slides with Vectashield (Vector Laboratories). Pupal wing images were acquired at room temperature using a confocal microscope (40 × ∼ oil immersion, 1.4 NA; SP5 DMI; Leica) with LAS AF (Leica) software. Images were processed with ImageJ (National Institutes of Health) and Photoshop (CS4; Adobe).

Mouse anti-Fmi (1:10; DSHB) and anti-Stbm (1:1000, gift form David Strutt) were used as primary antibodies. Rhodamine-phalloidin (1:500; Invitrogen) was used as a primary antibody for actin filaments. Fluorescent secondary antibodies were from Jackson Laboratories and Invitrogen (Alexa 568).

### Data availability

Strains and screening data are available upon request. The authors state that all data necessary for confirming the conclusions presented in the article are represented fully within the article.

## Results

### Expression of DmrD-Fz-GFP with different wing drivers is leaky

Research studies have used the accumulation of proteins in the ER to assess protein transport and trafficking in living cells for a long time. A suitable system is based on the use of the misfolded thermo-sensitive mutant of the viral capsid glycoprotein from the Vesicular Stomatis Virus (VSVG), called VSVG-tsO45. This protein accumulates in the ER at 40° and, after a temperature switch to 32°, the protein folds properly and is transported through the biosynthetic delivery pathway from the ER to Golgi/Endosomes, finally arriving at the plasma membrane (Doms *et al.* 1987; Hurtley and Helenius 1989; Musch *et al.* 1996; Presley *et al.* 1997). Several systems have since been developed to investigate these processes for any protein, following the same concept of protein accumulation at the ER. These include the DmrD-system (Rivera *et al.* 2000) or retention using selective hooks (RUSH) system (Boncompain *et al.* 2012). The DmrD system, first developed by ARIAD (and commercialized by Clontech), is based on accumulation of the respective fusion protein in the ER due to the dimerization domain (DmrD), which causes aggregation of the protein (Figure S1). The release from the ER in this case is caused by the addition of a drug, D/D solubilizer (from Clontech), which competes with the interaction between the DmrDs leading to disaggregation of the respective chimeric protein (Figure S1). Subsequently, during processing in the biosynthetic delivery pathway, DmrD domains are removed from the chimeric protein by furin cleavage in the Golgi, releasing the protein of interest in its native form (Figure S1).

**Figure 1:**
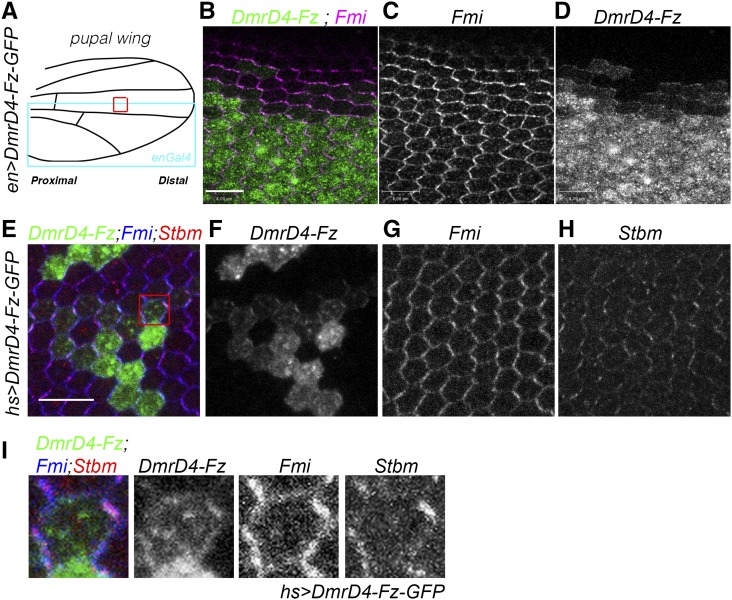
Expression of DmrD-Fz-GFP with different drivers in the wing is leaky. (A) Schematic illustration of a pupal wing highlighting the *engrailed* (*en*)*-GAL4* expression domain (blue box) and the wing area imaged in panels (B–D) (red box). (B–D) *en*-driven expression of *DmrD4-Fz-GFP* without D/D solubilizer is able to deliver Fz-GFP to cellular junctions, where it colocalizes with Fmi [magenta in (B) and monochrome in (C)] in cells expressing low levels of DmrD4-Fz-GFP at the border between the anterior and posterior (*en*) wing compartments [gray in (B) and monochrome in (D)]. Bar represents 8 μm. (E–I) Similar DmrD4-Fz-GFP protein delivery to cell-cell contacts is observed using *GAL4* under the control of a heat-shock promoter. Heat-shock for 6 hr or less produces clonal low levels of expression of DmrD-Fz-GFP in pupal wing cells, leading to Fz-GFP [green in (E) and monochrome in (F)] colocalization with Fmi [blue in (E) and monochrome in (G)] and Vang/Stbm [red in (E) and monochrome in (H)]. (I) Higher magnification of a low level expression DmrD-Fz-GFP cell showing colocalization of Fz-GFP, Fmi, and Vang/Stbm. Bar represents 10 μm. GFP, green fluorescent protein.

We have recently used this system in live tissue, in *Drosophila* third instar wing discs, to address the function of Arf1 in the biosynthetic delivery of the core PCP protein Fz (Carvajal-Gonzalez *et al.* 2015). We observed that the DmrD system does not generate a complete block of protein transport in the ER, and indeed a small proportion of the (initially chimeric) Fz protein was detected at the plasma membrane before the D/D solubilizer was added to the culture media (Carvajal-Gonzalez *et al.* 2015). Following expression of the respective Fz fusion protein at later stages of wing development, we noted that leakiness of the system was also detected in *Drosophila* pupal wings, where expression of DmrD-Fz-GFP was driven using different *GAL4* drivers, *e.g.*, *nubbin-GAL4* (*nub-Gal4)* (Figure S2), *engrailed-GAL4* (Figure 1, A–C), or using *GAL4* under the control of a *heat-shock* promoter induced for only 5–6 hr (Figure 1, E–I). In cells where the expression was moderate/low, we were able to detect Fz-GFP in perfect colocalization at the cellular junctions with Fmi and Vang/Stbm, and, in addition, in other intracellular organelles (Figure 1 and Figure S2).

Taken together, these experiments demonstrate that the DmrD system does not completely block the transport of chimeric DmrD-Fz-GFP to the cellular junctions, where it can colocalize with the other core PCP members.

### Expression of DmrD-Fz-GFP generates a GOF phenotype

**Figure 2:**
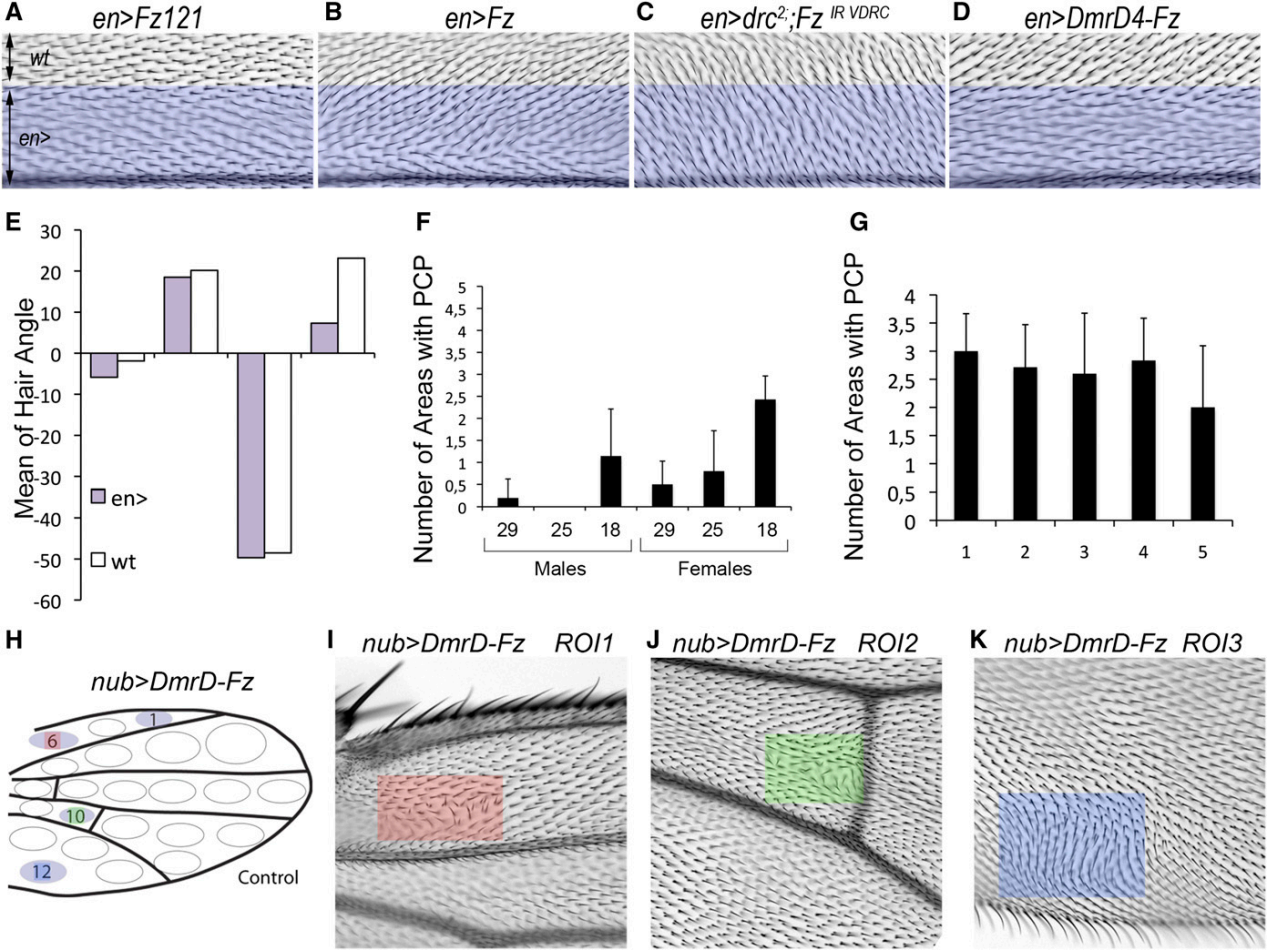
Expression of DmrD-Fz-GFP generates a localized gain-of-function phenotype. (A–D) Both overexpression (B) and knock-down of Fz (C) generate PCP phenotypes in the *en*-expression domain (posterior wing compartment), which is not observed with a nonfunctional PCP Fz [(A); Fz121, contains the extracellular and intracellular domain of Fz1 and the transmembrane domains of *Drosophila* Fz2, described in Wu *et al.* (2004)]. In addition, nonautonomous phenotypes are present in the adjacent cells of the anterior compartment, neighboring the expressing cells in both Fz GOF and LOF. DmrD-Fz-GFP overexpression produces a phenotype similar to the Fz overexpression (GOF) phenotype [compare (B) and (D)], and opposite to the Fz knock-down/LOF phenotype [compare (C) and (D)]. (E) Hair angle quantification for *en*-driven expression of Fz121, Fz, *fz*-IR, and DmrD-Fz-GFP in the anterior (white bars) and posterior compartment (purple bars), also showing that *en > DmrD-Fz-GFP* overexpression is manifest in a phenotype similar to Fz GOF wings. Bars represent mean of the angles for each genotype and wing compartment. (F) Quantification of the number of areas with PCP defects in *nubbin*-driven expression of *DmrD4-Fz-GFP* in males and females. At different temperatures (29, 25, and 18°) males and females generated different severity in PCP phenotypes. (G) Number of wing areas with PCP defects in *nub > DmrD4-Fz-GFP* females at 18° in five different experiments (performed over 6 months). (H) Quantification for each wing area (depicted by elliptical outlines) with PCP phenotypes quantified in 10 wings from 10 *nub > DmrD4-Fz-GFP* females at 18°. (I–K) Examples of adult wing areas where PCP defects are often observed close to the hinge between L1 and L2 [(I) and highlighted by red square], close to the posterior cross vein between L4 and L5 [(J) and highlighted by green square], and in the most posterior part of the wing between the posterior border and L5 vein [(B) and highlighted by blue square]. Each bar represents average wing areas with PCP defects for each genotype, and error bars represent SD. GFP, green fluorescent protein; GOF, gain-of-function; LOF, loss-of-function; PCP, planar cell polarity.

Next, we wanted to examine if this additional/increased accumulation of Fz-GFP at the cellular junctions, and repolarization of Fmi, could induce PCP phenotypes in adult wings due to a Fz GOF or LOF effect. It is well-established that both Fz GOF and LOF can generate PCP defects in the wing (Krasnow and Adler 1994; Vinson and Adler 1987). In this particular case, since we were overexpressing Fz and retaining it mostly in the ER, we wanted to test if the observed phenotype was a GOF (because flies have more Fz) or a LOF (because Fz was sequestering other core PCP players in the ER), where the extra Fz-GFP was trapped. To distinguish between these two possible scenarios, we tested for nonautonomy in wings overexpressing *DmrD-Fz-GFP* in the posterior compartment employing the *GAL4*/*UAS* system with *engrailed-GAL4* as driver (Figure 2). As established, overexpression of a chimeric Fz1/Fz2 protein (Wu *et al.* 2004), which is unable to signal in the PCP process, does not generate PCP defects in the posterior domain or neighboring cells (Figure 2A, and quantified in Figure 2E). On the contrary, wild-type Fz overexpression in the posterior domain caused hair orientation defects within the domain, but also induced neighboring cells to orient their actin hairs/trichomes to point away from the expression domain (Figure 2B, quantified in Figure 2E). The reverse phenotype is observed when Fz is knocked-down in the posterior compartment, causing wild-type neighboring cells in the anterior compartment to orient toward the *en > Fz-IR* domain (Figure 2C, quantified in Figure 2E). When the *en > DmrD-Fz-GFP* effect was assessed, actin hairs/trichomes from anterior neighboring cells were pointing away relative to the posterior expression domain, similar to the phenotype observed with wild-type Fz overexpression (Figure 2C, quantified in Figure 2E; although actin hairs within the expression domain did not show reorientation). We conclude that, at low levels, overexpression, of DmrD-Fz-GFP was causing a GOF phenotype.

Together with the DmrD-Fz-GFP localization, these experiments confirmed that *en* > *DmrD-Fz-GFP* flies displayed PCP phenotypes due to the leakiness of the retention system in cells that do not express high enough DmrD-Fz-GFP levels to cause successful aggregation in the ER. Additionally, we did not observe a depletion or delocalization of Fmi or Vang/Stbm at the plasma membrane upon *DmrD-Fz-GFP* overexpression, which would reflect a LOF effect (Figure 1 and Figure S1).

### Nubbin-driven DmrD-Fz-GFP serves as a good background for a screen

Based on these observations, we decided to further examine wings from adult flies expressing the construct without drug treatment at different temperatures, modulating the *GAL4/UAS* activity and testing for possible PCP phenotypes. We collected and scored adult wings from males and females expressing *DmrD-Fz-GFP* driven by *nubbin-GAL4* in the absence of the D/D solubilizer at 18, 25, and 29°. Close analyses of those wings revealed an increase in wing areas with PCP defects, including mch and hair orientation defects, which correlated with a decrease in temperature (Figure 2F); Males and females showed more severe PCP defects at lower temperatures as compared to higher temperatures. These results further support the conclusion that lower expression levels produce insufficient DmrD-Fz-GFP protein to efficiently trap it at the ER. In addition, male wings produced weaker PCP phenotypes as compared to female wings at the same temperature (Figure 2F). The PCP phenotypes were generally localized to two to three regions in the wing on either side of the wing blade (Figure 2, H–K).

Taken together, the localized phenotypic defects in adult wings of *DmrD-Fz-GFP* driven by *nubbin-GAL4*, the reproducibility of this Fz GOF PCP phenotype (Figure 2G), and the mild severity of the phenotype at low temperatures, prompted us to conduct a modifier screen. Based on our experience, low temperature allows good survival of the flies at later stages in the screening process, when RNAi lines are used. In addition, localized phenotypes increase reproducibility, minimizing the error in the scoring system (Figure 2G).

### Nubbin-Gal4-driven DmrD-Fz-GFP is a modifiable genetic background

A key element for a modifier screen is to actually be able to alter the observed phenotypes by gene dosage reduction. Although the temperature experiments presented above and differences between female-male wings (Figure 2F) already support that characteristic, we first tested known PCP mutants for their interaction with the *nub > DmrD-Fz-GFP* background at 18°. We tested *dsh^V26^*, *dgo^380^*, *pk-sple^6^*, *fz^p21^*, *aPKC*^-^, and several *scrib* mutants (reviewed in (Adler 2002; Klein and Mlodzik 2005; Seifert and Mlodzik 2007; Strutt 2003; see also Courbard *et al.* 2009; Djiane *et al.* 2005) in combination with *nub > DmrD-Fz-GFP* at 18° in females. We choose females and 18° because we could observe both positive and negative interactions with the respective phenotypic baseline of the PCP defects (Figure 2, H–K).

**Figure 3:**
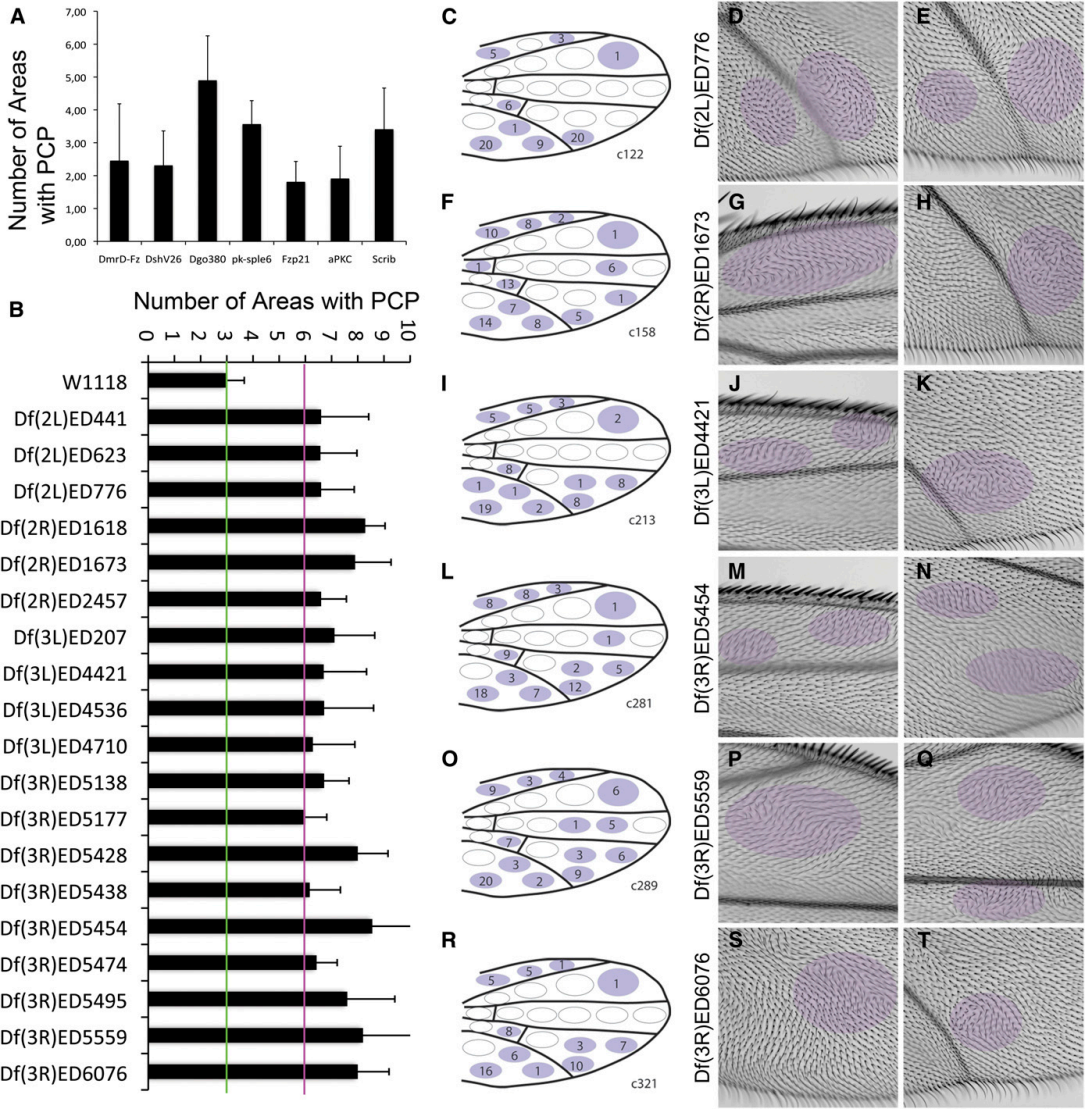
Screen for dominant modifiers of *nub > DmrD4-Fz-GFP*. (A) Genetic interactions of the *nub > DmrD-Fz-GFP* genotype and commonly used PCP alleles quantified in *nub > DmrD4-Fz-GFP* females at 18°. Each bar represents average wing areas with PCP defects for each genotype and error bars represent SD. (B) Histogram showing all deficiencies with an average increase of at least six areas per wing with PCP defects detected in the screening. (C–T) Schematic representation of phenotypes observed and two example images for 6 of the 19 interacting DrosDel deficiencies presented in (B). Compare schematic illustration to (H) in Figure 2. The number inserted in each area represents the number of times the respective area displayed PCP phenotypes. Areas with PCP defects are highlighted by purple overlay. GFP, green fluorescent protein; PCP, planar cell polarity.

Our pilot screen showed that *dgo*, *pk-sple*, and *scrib* were able to modify the baseline phenoptype. *pk-sple* and *scrib* showed a reproducible increase of 1.5 affected areas per wing, whereas *dgo* showed an increase of 2.5 affected areas per wing (Figure 3A). No clear interactions were observed with *fz^P21^*, *dsh^V26^*, or *aPKC* mutants (Figure 3A). This lack of interaction with *dsh* or *aPKC* could be due to the specific mechanism by which the mild GOF phenotype is achieved under these conditions. One possibility might also be that components of the proximal complex could be more sensitive or the severity of individual PCP mutant alleles used for the interactions. We concluded that the *nub > DmrD-Fz-GFP* background presented a modifiable phenotype suited for a screen.

### Deficiency screen

Several genetic modifier screens looking for new PCP regulators have been successfully performed in the past (*e.g.*, Rawls and Wolff 2003; Strutt and Strutt 2003; Weber *et al.* 2012) Using the *nub > DmrD-Fz-GFP* background and a similar strategy, we decided to ask whether we can identify additional regulators or effectors of Fz. As this Fz GOF phenotype is easy to score, it allowed us to perform a genome-wide screen with the *Drosophila* deficiency (Df) collection and to look for both suppressor and enhancer-type interactions.

We screened the DrosDel deficiency collection (from Bloomington), covering the second, third, and fourth chromosomes, testing for dominant modifiers. The fact that we could screen in adult wings made it efficient, as no immediate histological analyses were needed for the screen, and wings could simply be observed under a microscope. Since homozygous *nub > DmrD-Fz-GFP* flies are viable, we collected female virgins and crossed them with males from the DrosDel Df collection. In the primary screen, we mounted and examined the cellular hair/trichome appearance in 10 adult wings per genotype (in more than 90% of the crosses), quantifying the effects via the number of areas per wing affected on either side of the wing blade (see scheme depicted in Figure 2H). A total of 279 deficiencies were screened in this manner, covering nearly 80% of the genome. Of these, 19 large deficiencies (6.8% of tested) enhanced the wing PCP phenotype of the genetic background, by doubling the number of areas affected (from three areas in the parental genotype to at least six affected areas in the respective wings; Figure 3 and Table 1). Among the positive Df hits, we found that *Df*(*2R*)*ED1618* and *Df*(*2R*)*ED1673* overlap partially, and Df(3R)ED5428 covers Df(3R)ED5438 and Df(3R)ED5454, which left 16 nonoverlapping genomic regions. Fifteen percent showed a PCP increase of two areas per wing and 87.5% of the deficiencies did not show an effect (only an increase or decrease of one area or none per wing). Interestingly, two Df were able to almost fully suppress the Fz-GOF phenotype (Df(3L)ED4457 and Df(3R)ED5942).

**Table 1:** DrosDel deficiencies with an average of six wing areas with PCP phenotypes, detail on the respective smaller deficiencies, the number of genes tested for each genomic region, and gene/s identified to be responsible for the genetic interaction

Df Number	Smaller Df	Potential Genes	Genes Identified
*Df*(*2L*)*ED441*		18	***sem1***
*Df*(*2L*)*ED623*			*dachs*
*Df*(*2L*)*ED776*	N/C		
*Df*(*2R*)*ED1618**^a^*			*pk*
*Df*(*2R*)*ED1673**^a^*			*pk*
*Df*(*2R*)*ED2457*			*rho1*
*Df*(*3L*)*ED207*		15	N/I
*Df*(*3L*)*ED4421*	*Df*(*3L*)*ED4414*	31	***mRpL12*, *CG13310***
*Df*(*3L*)*ED4536*		8	***Dichaete* (*D*)**
*Df*(*3L*)*ED4710*	N/F		
*Df*(*3R*)*ED5138*	N/F		
*Df*(*3R*)*ED5177*		7	***kra*, *atu***
*Df*(*3R*)*ED5428**^b^*			
*Df*(*3R*)*ED5438**^c^*			
*Df*(*3R*)*ED5454**^c^*	N/C		
*Df*(*3R*)*ED5474*	N/F		
*Df*(*3R*)*ED5495*	N/F		
*Df*(*3R*)*ED5559*	*Df*(*3R*)*Exel6161*	12	***CG14712***
*Df*(*3R*)*ED6076*	*Df*(*3R*)*Exel6188*	24	***mRpL35***

Newly identified genes are highlighted in bold. Df, *Drosophila* deficiency; N/C, nonconclusive smaller deficiencies; N/F, not followed with smaller deficiencies or RNAi lines; N/I, not indentified with RNAi lines.

a Df(2R)ED1618 and Df(2R)ED1673 overlap partially.

b Df(3R)ED5428 contains Df(3R)ED5438 and Df(3R)ED5454.

c Df(3R)ED5438 and Df(3R)ED5454 are identical in genes covered.

Out of the 19 deficiencies with an increase of three times the phenotype (Figure 3B), three contained known PCP factors, effectors, or regulators (Table 1). The overlapping deficiencies Df(2L)ED1618 and Df(2L)ED1673 contain *prickle/spiny legs*, Df(2L)ED623 contains *dachs*, and Df(2R)ED2457 covers *rho1*. This initial result further confirmed the premise of our screening set up.

### Refinement of large deficiencies and possible new PCP candidate regulators

By using smaller, subdividing, and overlapping deficiencies, we narrowed down the initial genomic region responsible for an interaction of five large deficiencies (*Df*(*2L*)*ED776*, *Df*(*3L*)*ED4421*, *Df*(*3R*)*ED5454*, *Df*(*3R*)*ED5559*, and *Df*(*3R*)*ED6076*). The same scoring of wing areas was used to refine the genomic region of interaction. Out of the five original deficiencies, we were able to narrow down the genomic region for three (see smaller deficiencies in Table 1). The other two were nonconclusive (Table 1 and Figure S3). This approach helped identify smaller regions that could be analyzed directly with transgenic *UAS-RNAi* lines.

We added selected genes to the RNAi secondary screen set for four additional deficiencies. In three of those four, we were able to narrow down the genomic region using the overlap with other large Drosdel Deficiencies (*Df*(*2L*)*ED441*, *Df*(*3L*)*ED207*, and *Df*(*3L*)*ED4536*). The fourth one was *Df*(*3R*)*ED5177*, which only contains seven genes. In total, we screened a total of 116 genes from seven different genomic regions (Table 1) using *UAS-RNAi* lines (listed in Table S1, Table S2, and Table S3).

We screened the siRNA lines using the *nub > DmrD-Fz-GFP* flies with the same scoring system that we used for the DrosDel deficiencies. Out of the 116 genes tested, we identified eight genes that showed a clear interaction with *nub > DmrD-Fz-GFP* (Table 1). Those were the mitochondrial proteins *mRpL12* and *mRpL35*, *Dichaete* (*D*), *CG14712*, *sem1*, *CG13310*, the elongation initiator factor *krasavietz* (*Kra/eIF5C*), and *atu*.

### Krasavietz and Shot as novel PCP effectors/regulators

**Figure 4:**
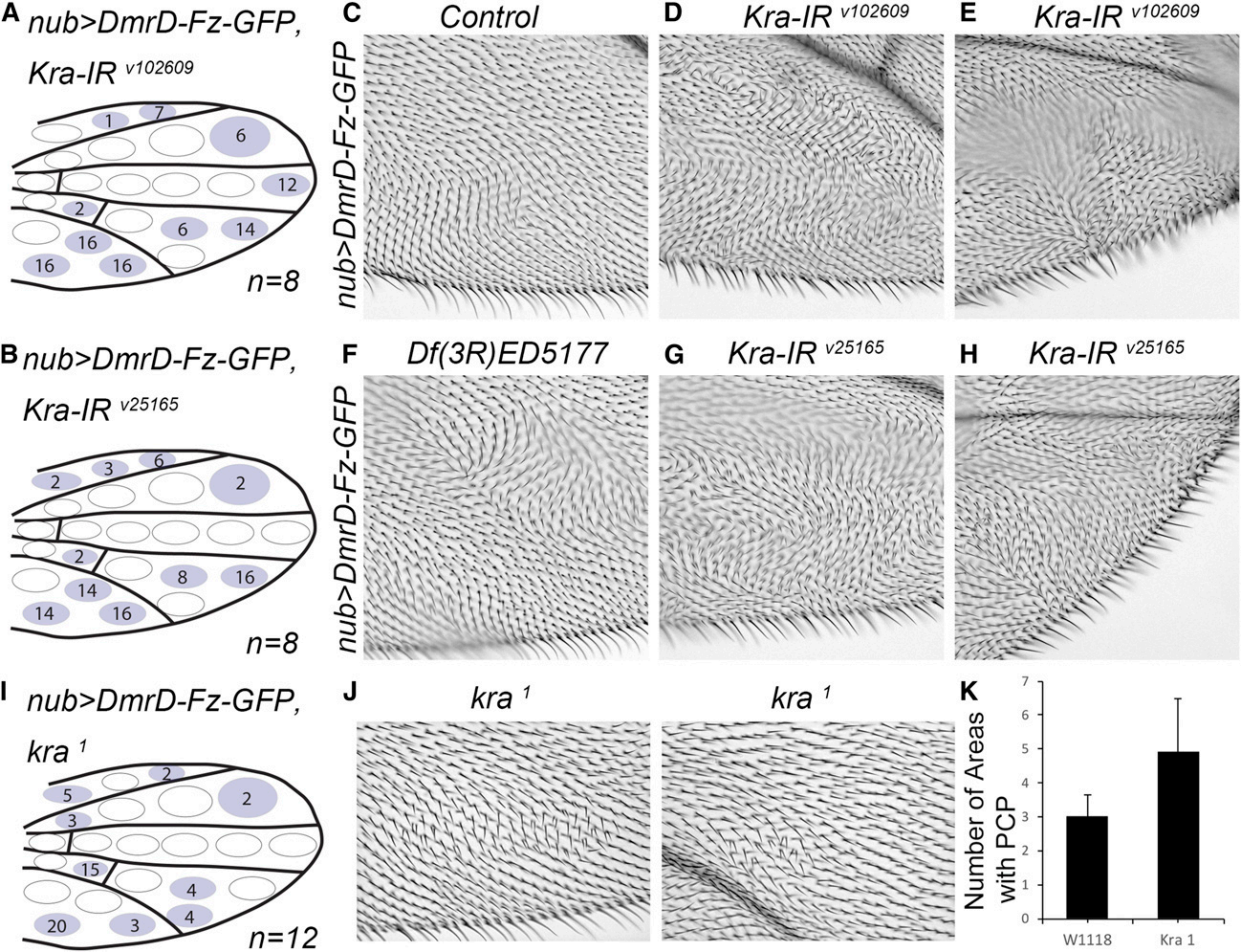
*kra* interactions with *nub > DmrD4-Fz-GFP* genotype. (A and B) Two independent *Kra*-IRs, v25165 (B) and v102609 (A), and the *kra^1^* mutant (I) enhanced the *nub > DmrD4-Fz-GFP* defects in females at 18°, shown schematically [compare (A), (B), and (I) to Figure 2H]. Numbers associated with each wing area represent the time PCP defects were observed for all wings collected and scored on the dorsal and ventral side. *n* represents the number of flies quantified for each genotype. (C) Control wing image for *nub > DmrD4-Fz-GFP* females (named control). (F) Example of the phenotype observed in Df(3R)ED5177. (D and E) Examples for *Kra*-IR v102609, and (G and H) *Kra*-IR v25165. (J and K) Examples for *kra^1^* mutant interactions and quantification (*w^1118^* is the control crossed to *nub > DmrD4-Fz-GFP* to establish the basal PCP phenotype levels). GFP, green fluorescent protein; PCP, planar cell polarity.

Of note is that *Df*(*3R*)*ED5177*, which covers only seven genes, contained two hits, *atu* and *kra* (Figure 4 and Figure S4). Revisiting the bibliography and FlyBase we found that Atu was a LEO-like protein that is part of the Paf1 complex. Interestingly, Kra is known to interact with the spectraplackin Short-stop (Shot) ( Lee *et al.* 2007), a linker between actin and microtubules in the cytoskeleton. Based on these published results, we decided to further confirm and characterize *kra* in the PCP context.

**Figure 5:**
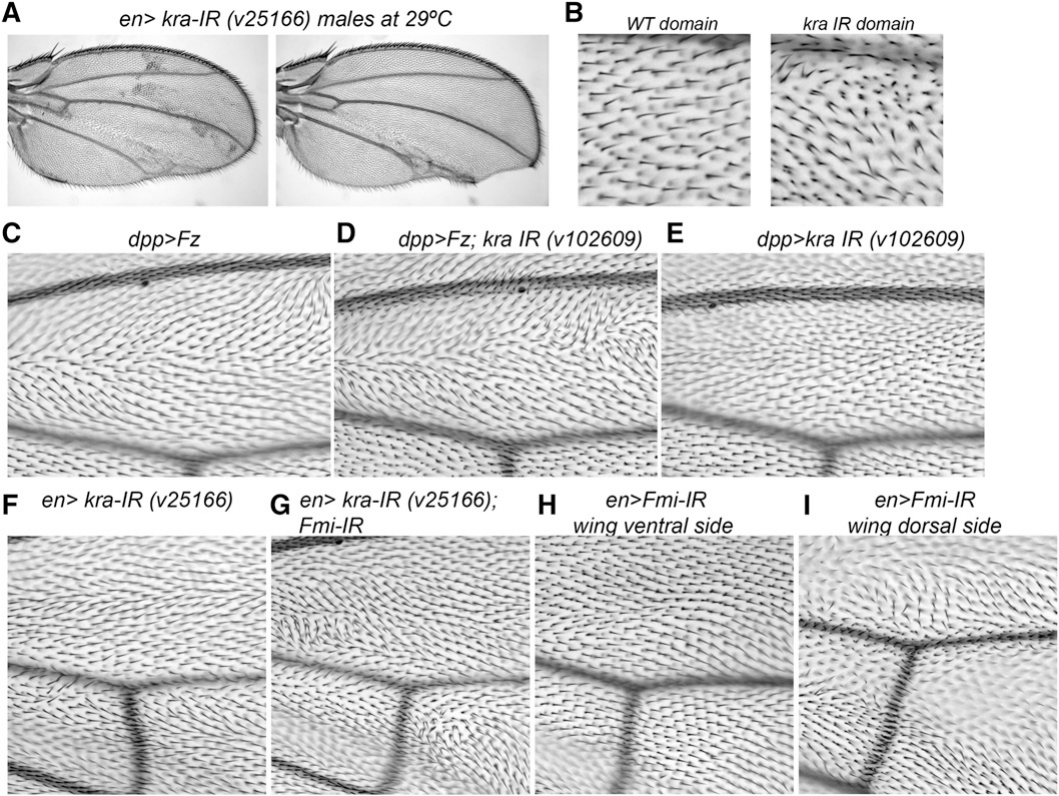
*kra* is a new PCP regulator. (A and B) Examples of *en*-driven *kra* knock-down with RNAi line v25166 at 29° displaying PCP-related phenotypes, including mch defects [high magnification in (B) of a WT domain or a *kra*-IR from the same wing] and tissue loss. Hair orientation defects were difficult to score due to growth defects in the posterior compartment; wing margin defects were also observed. (C–E) Kra interaction with core PCP factors was confirmed, using a different Fz overexpression system used in previous studies (Jenny *et al.* 2005). Combination of *Kra*-IR with *dpp > Fz* showed a strong increase in mch defects, as compared to *dpp > Fz* alone [compare (D) and (C)]. No phenotype was observed in *dpp > Kra-IR* alone under these conditions [(E), as control], confirming an interaction. Combination of *Kra-IR* with *en > Fmi-IR*, a PCP loss-of-function genotype, caused an increase in mch defects, as compared to *en > Fmi-IR* alone [compare (F) and (G)]. No phenotypes were observed in *en > Kra-IR* alone under these conditions, at 18° (H). Similarly, no defects were detected at 18° in this ventral wing surface in *en > Fmi-IR* flies, but PCP phenotypes were imaged in the DORSAL side of such wings (I). PCP, planar cell polarity; RNAi, RBNA interference; WT, wild-type.

We confirmed that a *kra* mutant on its own enhanced the *nub > DmrD-Fz-GFP* phenotype (Figure 5, I and J). We also confirmed the interaction of *Kra-IR* and a known Fz GOF phenotype, using wild-type Fz overexpression under *dppGAL4* control (Jenny *et al.* 2005). We found that while *dpp > Kra-IR^v102609^* by itself did not show a clear phenotype (Figure 5E), the combination of *Kra-IR* with *dpp > Fz*, enhanced the Fz GOF phenotype (Figure 5C), and most strikingly caused a dramatic increase of cells with multiple hairs (Figure 5D), a phenotype associated with PCP- (de) regulated actin polymerization.

In addition, we tested the *Kra* interaction with a PCP LOF genotype. In this case, we knocked-down Fmi, a core Fz-PCP factor and interactor of Fz, via *engrailed*-driven expression of *Fmi-IR*. *en > Fmi-IR* showed hair/trichome orientation defects in both sides of the wing blade in the posterior compartment, but this was less obvious in the proximal part of the wing close to the cross veins in the ventral side (Figure 5, H and I). We tested the interaction in this region of the wing. The combination of *Kra-IR* with *en > Fmi-IR* produced mch phenotypes (Figure 5G) that were otherwise never observed in this wing region with either *en > Fmi-IR* (Figure 5H) alone or *en > Kra-IR* (Figure 5F). Both experiments independently confirmed a role of Kra in PCP establishment by genetically interacting with Fz and Fmi.

To test if Kra could generate PCP-associated phenotypes by itself, we tested *Kra-IR* lines by themselves at higher temperatures (as compared to the screen). We tested individual lines by themselves, and although two of them showed no phenotypes with *engrailed-Gal4*, *Kra-IR ^v25166^* displayed PCP-related phenotypes at 29° (Figure 5, A and B). In the posterior compartment, *Kra-IR ^v25166^* showed, besides growth defects, multiple cellular hairs (Figure 5, A and B). In addition, we tested RNAi lines for the remaining gene set of screen hits (Sem1, mRpL35, mRpL12, CG13310, D, and CG14712). For most of them, *engrailed*-driven expression at three different temperatures produced lethality. Only CG14712 and CG13310 were viable when knocked-down under *en-Gal4* and generated wing margin defects, notches, and blistering (Table S4).

**Figure 6:**
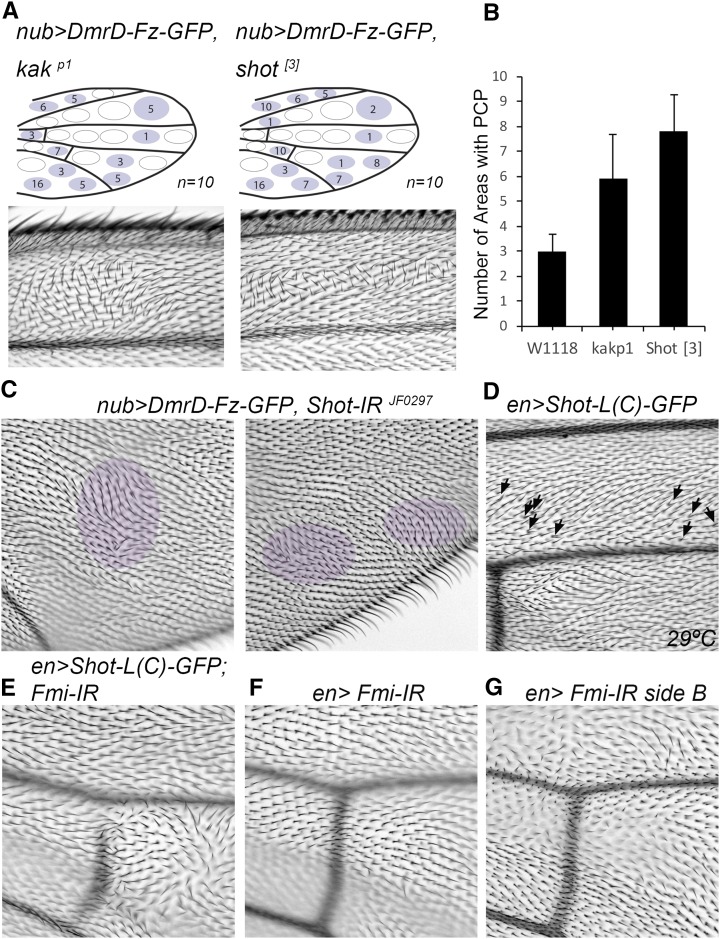
*Shot* knock-down and overexpression enhances core PCP phenotypes. (A and B) Combination of *nubbin*-driven DmrD-Fz-GFP with *shot* mutants (*kak^p1^* and *shot*[3]) enhanced the PCP defects when compared to control conditions. (C) High magnification images of posterior regions of *nub > DmrD-Fz-GFP*, *>Shot-IR* wings displaying defects in cellular hair number (mch) and orientation. (D) Shot overexpression under *en-Gal4* control causes mch phenotypes in the posterior domain of the wing. (E–G) Combination of Shot-GFP and *en > Fmi-IR* shows a marked increase in PCP defects in the ventral side of the wing; no such phenotypes are detected in *en > Fmi-IR* alone (F) or Shot-GFP alone. GFP, green fluorescent protein; PCP, planar cell polarity.

Due to a reported functional link between Kra and Shot (a.k.a. *kakapo*) (Lee *et al.* 2007), we decided to test *nub > DmrD-Fz-GFP* also for an interaction with Shot. It is well-established that *shot* mutants produce wing blistering (Prout *et al.* 1997), a phenotype we also detected in *dpp*-driven *Shot-IR* flies (Figure 6A). When we assessed *Shot-IR* in our screening system in combination with *nub > DmrD-Fz-GFP*, we observed a phenotype that was difficult to score following the same wing area scoring system as used in the screen (see above). In all flies, at least one third of the wing presented an inflated wing appearance that made it difficult to mount them without affecting the hair pattern. Nonetheless, in most wings we detected, in distal wing parts away from the inflated areas, patches of hairs with PCP phenotypes and both PCP orientation and number/mch defects (Figure 6C). To further test the interaction, we assayed *shot* mutants in combination with *nub > DmrD-Fz-GFP*. In this case, an enhancement of the PCP phenotype was observed similar to the one found with *kra* (Figure 6, A and B). In addition, we tested Shot overexpression using *en-Gal4* and obtained a mild PCP phenotype with some mch-type defects (Figure 6D). We also tested the interaction of Shot-overexpression with Fz and Fmi. Although we did not detect an interaction with Fz, we observed robust interactions with Fmi, similar to the effects of Kra (Figure 6, E–G). Taken together, we uncovered *kra* and *shot* as new PCP regulators/effectors, although further studies will be necessary to molecularly link Kra and Shot to the core PCP members.

## Discussion

Here, we describe a novel genetic screen for regulatory factors linked to Fz/PCP signaling. We have used a recently published Fz overexpression method, in which Fz is aggregated and accumulated in the ER through a self-aggregation domain (dimerization domain: DmrD) cloned in-frame (Carvajal-Gonzalez *et al.* 2015). Overexpression of this chimeric protein at low levels produces a mild PCP GOF phenotype as a result of leakiness of the aggregation system in the ER. We assayed the localization of *DmrD-Fz-GFP* under different conditions and drivers and observed that, in cells where *DmrD-Fz-GFP* is expressed at low levels, Fz is not retained in the ER as efficiently and thus is mildly increased at the plasma membrane (Figure 1). Under these conditions, Fmi and Vang/Stbm are overrecruited, affecting PCP; thus, this overexpression is able to reorganize the polarity of PCP complexes resulting in PCP defects (Figure 1 and Figure 2).

Taking advantage of the *DmrD-Fz-GFP* flies and their behavior, we have performed a genetic screen using the Drosdel Df collection, and identified at least 19 large deficiencies able to strongly modify the control phenotype. Furthermore, using a combination of smaller deficiency and transgenic RNAi strains to identify new potential PCP regulators, we narrowed these regions down to 116 genes, and subsequently confirmed Kra and Shot as new “PCP regulators/effectors.”

All of the newly identified genes belong to functional categories that may provide new insight into PCP regulatory mechanisms. Regarding the mitochondrial ribosomal proteins L12 and L35 (mRpL12 and mRpL35), it is known that mRpL12 mutants produce cell growth defects due to the requirement of this protein by CycD/Cdk4 (Frei *et al.* 2005). In addition, mRpL12 mutants and overexpression flies display rough eyes (Frei *et al.* 2005) and mitochondrial organization defects (Frei *et al.* 2005). Similarly, mRpL35 has been shown to affect epithelial development upon mRpL35 knock-down, leading to the formation of tube-like ovarioles (Berns *et al.* 2014). Also, the identification of mitochondrial proteins mRpL12 and mRpL35 in a PCP signaling context is intriguing because PCP and mitochondrial function have been recently associated in *Drosophila*: Fat-PCP signaling interconnecting with both the Hippo pathway and mitochondrial activity (Sing *et al.* 2014).

The highly evolutionarily conserved Sem1 gene (Human homolog *DSS1*) is a 26S proteasome subunit (Paraskevopoulos *et al.* 2014; Sone *et al.* 2004; Tomko and Hochstrasser 2014) and, in vertebrates, SEM1/DSS1 bind to the tumor suppressor BRCA2 (Marston *et al.* 1999). SEM1 protein is part of the nuclear pore complex (Faza *et al.* 2009), but is also a regulator of the exocyst complex in yeast (Jantti *et al.* 1999). The exocyst complex is an essential multicomplex mediating polarized secretion. We have recently found that the trafficking machinery associated with Rab11 affects trichome formation (Gault *et al.* 2012). Exocyst protein Sec15 is a downstream effector of Rab11. Further experiments will be needed to dissect the *Sem1* phenotype in PCP and dissect which of these SEM1 functions are critical for PCP signaling. Interestingly, proteasome degradation through E3 ubiquitin ligases and PCP signaling have been linked in several studies, some of which have suggested that they affect Prickle1 stability in vertebrates (Narimatsu *et al.* 2009).

Much less is known for *CG14712*, *CG13310*, and the transcription unit *Atu*. *Atu* contains a Leo1-like protein domain and LEO1 is a well-established component of the PAF1 complex in vertebrates. Interestingly, Wnt signaling is directly connected to the PAF1 complex via armadillo/β-catenin and thereby controls transcription (Mosimann *et al.* 2006). In addition to the interaction between Atu and DmrD-Fz-GFP, we observed a mix of PCP and canonical Wnt signaling-associated phenotypes in *Atu*-IR conditions, including small wings, wing margin defects, and mch (Figure S4). These results might be reminiscent of Atu function in vertebrates, associated with the PAF1 complex and β-catenin.

The most straightforward hit was *krasavietz* (*kra*), an evolutionarily conserved putative translation factor, working as a translation inhibitor. Kra is a well-known interactor, both genetically and molecularly, of Short-stop (Shot, a.k.a. *kakapo*), which has a linker function between the microtubule and actin cytoskeleton, both in flies and vertebrates (Lee *et al.* 2007; Sanchez-Soriano *et al.* 2009). In flies, this interaction is required for cellular functions of Shot, for example supporting midline axon repulsion (Lee *et al.* 2007), where *shot* and *kra* dominantly enhance the frequency of midline crossovers (Lee *et al.* 2007). More specifically, Shot and Kra interact in an actin-dependent process, like filopodia formation, during neuronal growth (Sanchez-Soriano *et al.* 2009). A similar function was described in mammals for ACF7, a Shot homolog, which coordinates the organization of F-actin and microtubules to support the motility of neuronal growth cones [reviewed in Prokop *et al.* (2013)]. In addition, ACF7 is considered to be an epidermal plakin that integrates actin and microtubule networks at cellular junctions of epithelial cells (Karakesisoglou *et al.* 2000), and Shot is functionally linked to epithelial cells during tubule formation (Booth *et al.* 2014).

Both *shot* and *kra* LOF produce a similar phenotype at the CNS midline. Here, we describe a similar scenario, where both *shot* and *kra* produce a reproducible/similar interaction with DmrD-Fz-GFP, which was further confirmed for *kra* with Fmi. Interestingly, Shot overexpression also produces mch phenotypes and enhances the *fmi*-IR defects. Thus, we hypothesize that the function of *kra* and *shot* within PCP establishment is due to their regulatory function(s) at the level of the actin and microtubule cytoskeleton. This could affect junction maintenance among other roles and, thus, core PCP factor localization, or it could reflect their function as PCP effectors. Importantly, a genetic screen for regulators of dendritic outgrowth, branching, and routing in *Drosophila* identified *shot* and *fmi* together with several other genes (Gao *et al.* 1999). This might reflect additional connections between Shot and core PCP factors in nonepithelial contexts.

In summary, our novel genome-wide screen has identified a set of new genes, falling into several categories and expanding the biochemical and cellular repertoire associated with PCP establishment in *Drosophila* and likely also in vertebrates. In particular, the identification of Kra and Shot as new potential linkers between PCP signaling and actin and microtubule cytoskeletal dynamics is exciting, and will open up new research avenues in PCP establishment.

## Supplementary Material

Supplemental Material
