# Supplementary material for: A Novel Frizzled-Based Screening Tool Identifies Genetic Modifiers of Planar Cell Polarity in Drosophila Wings
Source: G3 (Bethesda). 2016 Oct 11;6(12):3963–73. doi: 10.1534/g3.116.035535 (PMC5144966; doi:10.1534/g3.116.035535)
Supplement: Supplemental Material [file supp_6_12_3963__index.html]

A Novel Frizzled-Based Screening Tool Identifies Genetic Modifiers of Planar Cell Polarity in Drosophila Wings — Supplemental Material 

# A Novel Frizzled-Based Screening Tool Identifies Genetic Modifiers of Planar Cell Polarity in *Drosophila* Wings

## Supplemental Material for Carvajal-Gonzalez, *et al*, 2016

**Files in this Data Supplement:**

- Figure S1 - Biology of the Fz-fusion protein used in the screen. (.pdf, 405 KB)
- Figure S2 - Behavior of the Fz fusion protein. (.pdf, 508 KB)
- Figure S3 - Examples of mapping strategies of genomic subset regions within the initial large DrosDel deficiency screen hits. (.pdf, 233 KB)
- Figure S4 - Effects of *atu* on PCP signaling and wing development. (.pdf, 550 KB)
- Table S1 - List of genes and VDRC stocks tested for each gene for DrosDel deficiencies Df(2L)ED441, Df(3L)ED207 and Df(3L)ED4421. (.pdf, 419 KB)
- Table S2 - List of genes and VDRC stocks tested for each gene for DrosDel deficiencies Df(3L)ED4536, Df(3R)ED5559 and Df(3R)ED6076. (.pdf, 397 KB)
- Table S3 - List of genes, VDRC, and Bloomington stock center stocks tested for each gene for DrosDel deficiency Df(3R)ED5177. (.pdf, 380 KB)
- Table S4 - Phenotypes observed in engrailed driven RNAi lines.(.pdf, 396 KB)
