# Supplementary material for: A Novel Frizzled-Based Screening Tool Identifies Genetic Modifiers of Planar Cell Polarity in Drosophila Wings
Source: G3 (Bethesda). 2016 Oct 11;6(12):3963–73. doi: 10.1534/g3.116.035535 (PMC5144966; doi:10.1534/g3.116.035535)
Supplement: Supplemental Material [file supp_g3.116.035535_FigureS1.pdf]

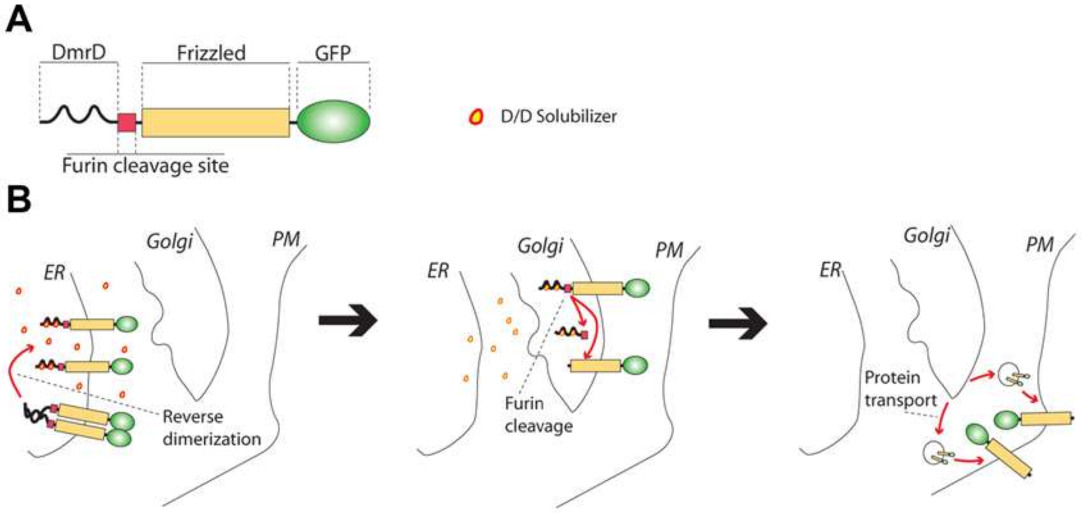

Figure S1: Biology of the Fz-fusion protein used in the screen

(**A**) Schematic representation of the chimeric Frizzled protein including the dimerization domain (DmrD) and the furin cleavage site (red) N-terminal to the Fz ORF. GFP was cloned in frame with Fz at the C-terminal. (**B**) Representation of the biosynthetic delivery pathway for DrmD-Fz-GFP in the presence of D/D solubilizer and highlights of the main processes in each step of the pathway. Addition of the D/D solubilizer produces disaggregates of the dimerization/multimerization allowing its transport from the endoplasmic reticulum to the Golgi. In the Golgi the chimeric DmrD-Fz-GFP is cleavaged by the Golgi resident protease Furin, generating two protein fragments, the DmrDs and Fz-GFP. From this point Fz-GFP follows its transport to the plasma membrane. D/D Solubilizer drug was not used in this study, as we relied on the leakiness of the system when expressed at lower levels. Fz-GFP delivered to the membrane is fully functional and can rescue *fz-* flies.
