# Supplementary material for: A Novel Frizzled-Based Screening Tool Identifies Genetic Modifiers of Planar Cell Polarity in Drosophila Wings
Source: G3 (Bethesda). 2016 Oct 11;6(12):3963–73. doi: 10.1534/g3.116.035535 (PMC5144966; doi:10.1534/g3.116.035535)
Supplement: Supplemental Material [file supp_g3.116.035535_FigureS2.pdf]

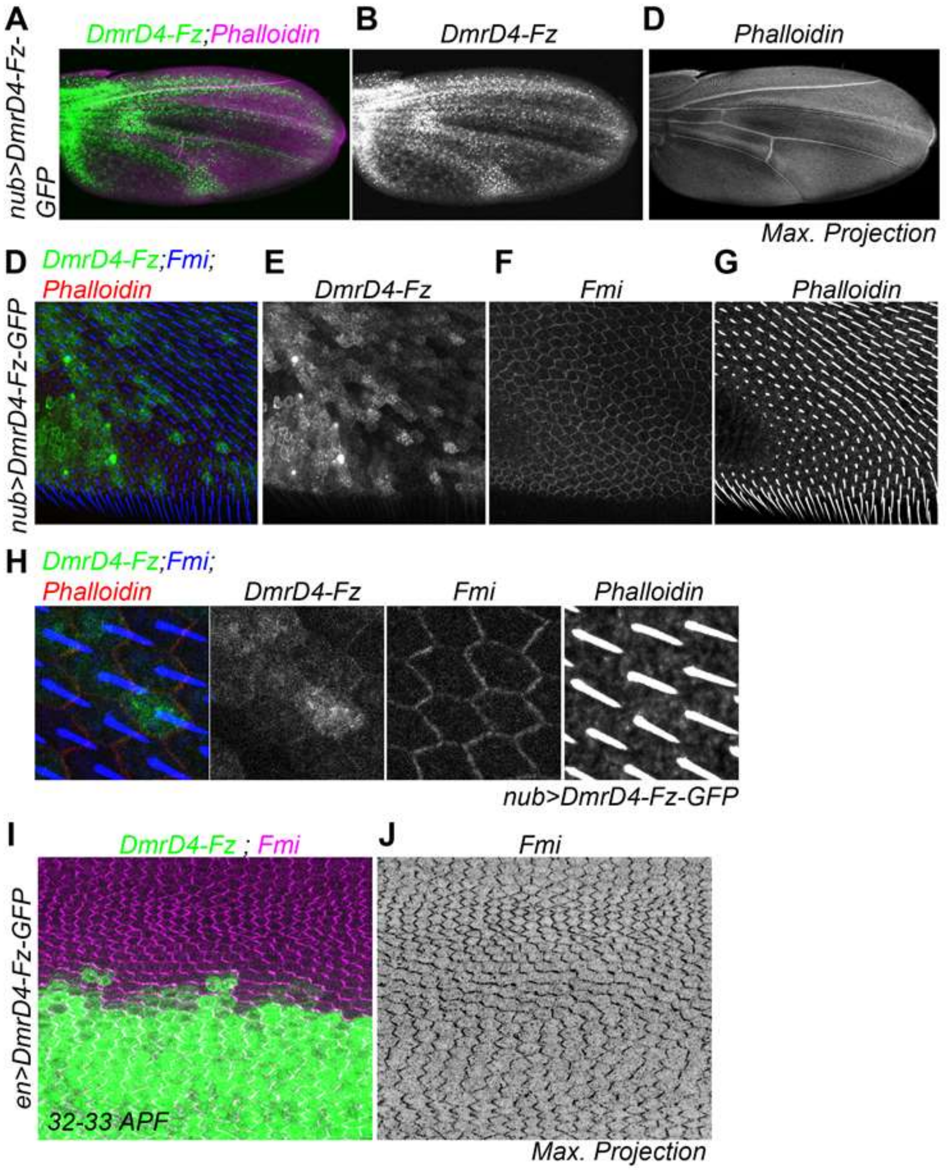

Figure S2: Behavior of the Fz fusion protein.

(**A**) *nubbin (nub)-Gal4* driven expression of *DmrD4-Fz-GFP* at 18°C (green in **A** and monochrome in **B**) without D/D solubilizer is uneven throughout the entire wing blade, labeled with phalloidin/F-actin (magenta in **A** and monochrome in **C**). Higher expression levels are detected in regions covering L2, L3, L4, L5 and both crossveins. (**D**) *nub*-driven expression of *DmrD4-Fz-GFP* at 18°C without D/D solubilizer its able to arrive to membrane junctions and colocalize with Fmi (red in **D,** monochrome in **F**) in cells expressing low levels of DmrD4-Fz-GFP (green in **D,** monochrome in **E**). Phalloidin marks F-actin (polymerized actin: blue in **D,** monochrome in **G**). (**H**) Higher magnification of a region from panel **D** with low levels of DmrD-Fz-GFP expression in cells, showing the colocalization of Fz-GFP and Fmi. (**I-J**) Low level *engrailed-Gal4* driven expression (18°C) of *DmrD4-Fz-GFP* without D/D solubilizer is able to reorient the localization of Fmi at the border between the anterior and the posterior compartment (magenta in **I**, monochrome in **J**). Scale bars represent 10μm.
