# Supplementary material for: A Novel Frizzled-Based Screening Tool Identifies Genetic Modifiers of Planar Cell Polarity in Drosophila Wings
Source: G3 (Bethesda). 2016 Oct 11;6(12):3963–73. doi: 10.1534/g3.116.035535 (PMC5144966; doi:10.1534/g3.116.035535)
Supplement: Supplemental Material [file supp_g3.116.035535_FigureS3.pdf]

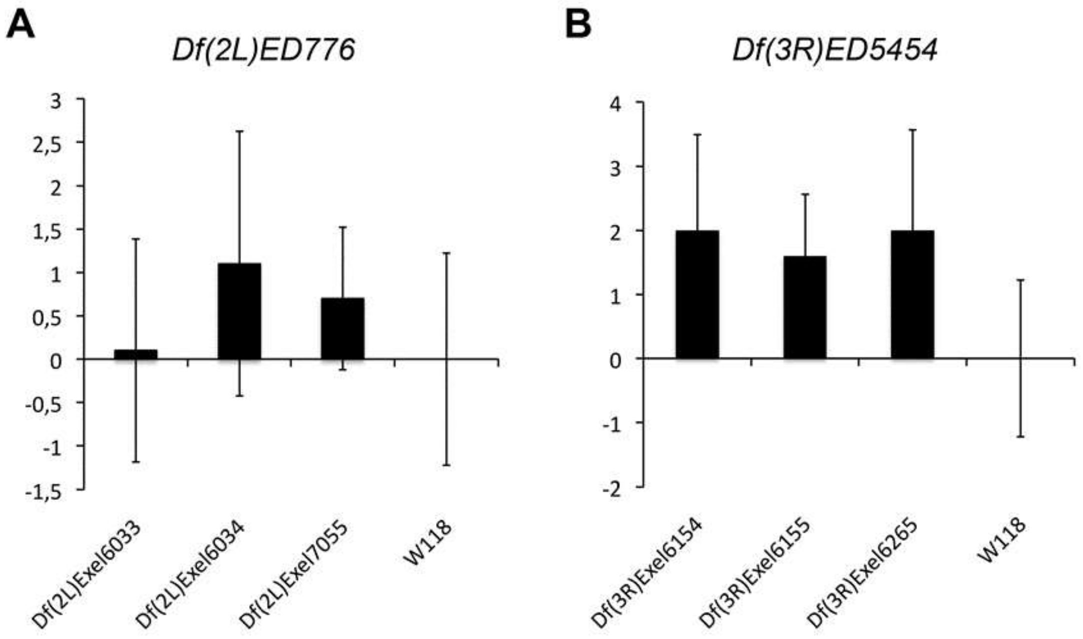

Figure S3: Examples of mapping strategies of genomic subset regions within the initial large DrosDel deficiency screen hits

(**A-B**) Smaller deficiencies used to narrow down the genomic region covered by large deficiencies Df(2L)ED776 (**A**) and Df(3R)ED5454 (**B**). Bars represent the average number of wing areas with PCP defects after subtracting the basal phenotype (3 areas per wing).
