# Supplementary material for: A Novel Frizzled-Based Screening Tool Identifies Genetic Modifiers of Planar Cell Polarity in Drosophila Wings
Source: G3 (Bethesda). 2016 Oct 11;6(12):3963–73. doi: 10.1534/g3.116.035535 (PMC5144966; doi:10.1534/g3.116.035535)
Supplement: Supplemental Material [file supp_g3.116.035535_FigureS4.pdf]

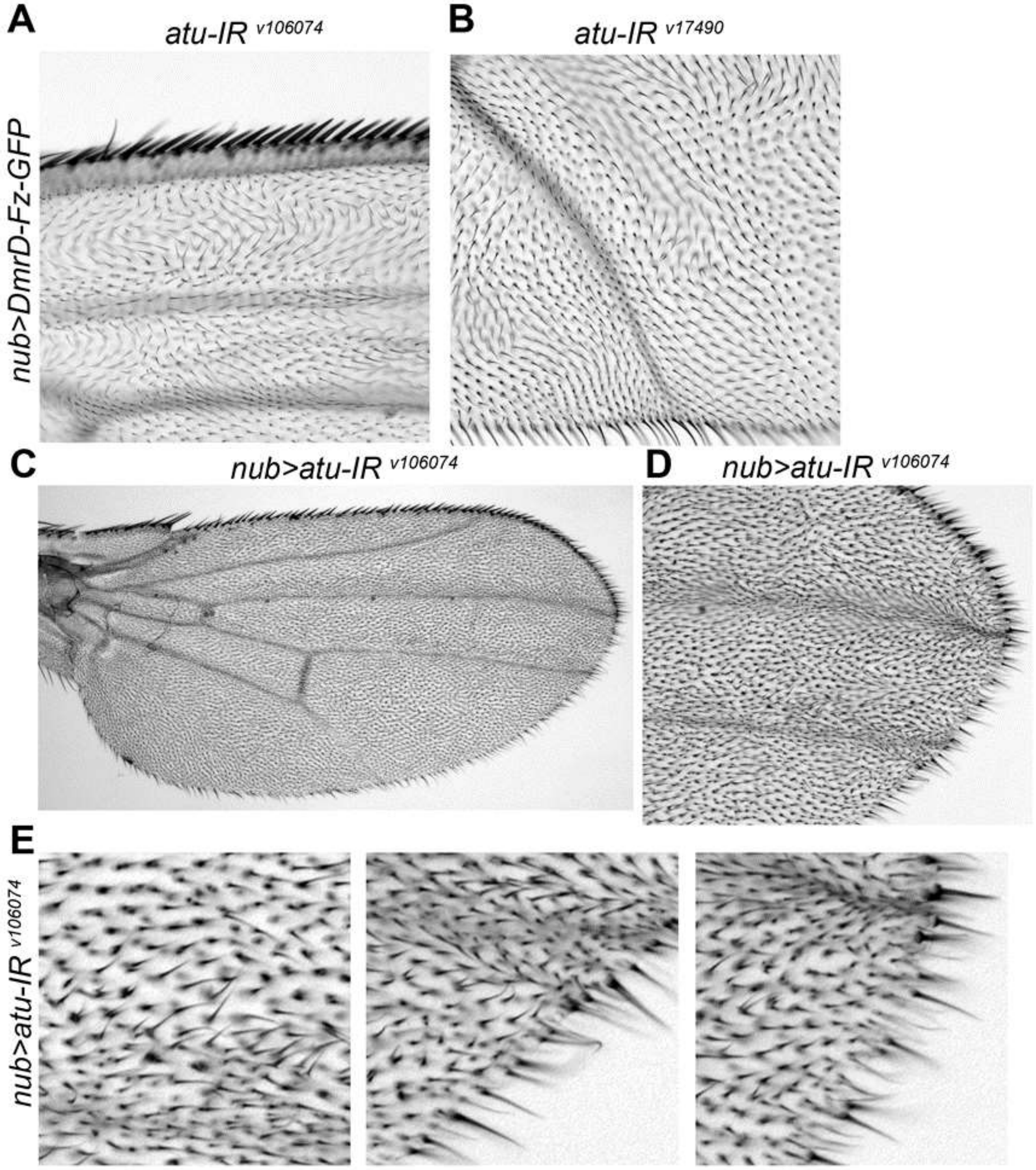

Figure S4: Effects of *atu* on PCP signaling and wing development.
(**A-B**) Two independent *atu-IR*, v106074 (**A**) and v17490 (**B**), enhanced the phenotype of *nub>DmrD4-Fz-GFP* females at 18°C (compare panels **A** and **B** to Figure 2H in main text). (**C-E**) *nub*-driven *atu* knock-down with RNAi line v106074 at 29°C generates PCP related phenotypes on itw own, including mch defects (**E**). Hair orientation defects/phenotypes were difficult to score due to general growth defects. In addition, small wings were recovered with significant margin defects (**C-E**).
