# Supplementary material for: A Novel Frizzled-Based Screening Tool Identifies Genetic Modifiers of Planar Cell Polarity in Drosophila Wings
Source: G3 (Bethesda). 2016 Oct 11;6(12):3963–73. doi: 10.1534/g3.116.035535 (PMC5144966; doi:10.1534/g3.116.035535)
Supplement: Supplemental Material [file supp_g3.116.035535_TableS1.pdf]

Table S1: List of genes and VDRC stocks tested for each gene for DrosDel deficiencies Df(2L)ED441, Df(3L)ED207 and Df(3L)ED4421.

**Df(2L)ED441**		**Df(3L)ED207**		**Df(3L)ED4421**	
**Gene Name**	**VDRC stock number**	**Gene Name**	**VDRC stock number**	**Gene Name**	**VDRC stock number**
ade3	v46293	***Cct1***	v100575	***CG13306***	v103049
	v46295		v18628		v44960
CG11266	N/A	***Cct2***	v105794		v44961
CG11289	v107242	***CG12003***	v102462	***CG13808***	v107895
	v45993		v51488		v13861
CG31907	v34019		v51925		v13862
CG31908	v51924	***CG12084***	v31646	***CG13309***	v101094
CG3476	v2734	***CG12090***	v110386		v14133
	v100372		v16390		v14134
CG43321	N/A	***CG12091***	v100800	***CG13310***	v101276
CG43322	v110475		v13985		v43959
Coprox	v105125		v13987		v43960
milt	v110193	***CG12099***	v109293	***CG13311***	v101655
	v41508		v18734		v51574
	v41752	***CG32313***	v104619	***CG13312***	v14060
	v47885		v23281		v14062
	v41507	***CG7852***	v103582	***CG13313***	v100332
	v41753		v7178		v5163
Mnn1	v110376	***CG7879***	v15260	***CG32023***	v108338
	v17701	***cue***	v104645	***CG32024***	v102205
Nuf2	v100235		v1043		v51580
	v23650	***Ir62a***	N/A		v52031
Pcp	v44901	***Pex10***	v110405	***CG32026***	v34032
	v44902		v46613	***CG34426***	N/A
Pvf2	v7628	***Psa***	v110657	***CG34427***	v100648
	v7629		v35354	***CG43169***	N/A
Rab30	v110230	***pUf68***	v109796	***CG5021***	v105709
	v27004		v20144		v36439
	v27002				v36440
smt3	v105980			***CG5026***	v105674
	v34113				v34915
Sem1	v107661				v34916
	v31787			***CG5644***	v102841
	v49153				v13002
	v31789				v13004
	v49152			***CG5653***	v104121
uif	v101153				v14064
	v1047				v14065
	v1050			***CG5660***	v107632
	v1051				v29445
					v29446

				CG5804	v23586
					v23587
				CG6576	v108232
					v26694
**Df(2L)ED441**		**Df(3L)ED207**		**Df(3L)ED4421**	
**Gene Name**	**VDRC stock number**	**Gene Name**	**VDRC stock number**	**Gene Name**	**VDRC stock number**
					v26695
				dally	v14136
				Fhos	v108347
					v13298
					v45836
					v45837
				GNBP3	v106591
					v37255
					v37256
				Mcm7	v106648
				mfr	v103726
				mRpL12	v100496
					v50149
					v26684
					v50150
				orb2	v107153
					v27498
					v27499
				pix	v44325
				Srp68	v104867
					v27351
				TrpA1	v37249
					v37250
				Tsp66E	v104430
					v37252
					v37253
