# Supplementary material for: A Novel Frizzled-Based Screening Tool Identifies Genetic Modifiers of Planar Cell Polarity in Drosophila Wings
Source: G3 (Bethesda). 2016 Oct 11;6(12):3963–73. doi: 10.1534/g3.116.035535 (PMC5144966; doi:10.1534/g3.116.035535)
Supplement: Supplemental Material [file supp_g3.116.035535_TableS2.pdf]

**Table S2:** List of genes and VDRC stocks tested for each gene for DrosDel deficiencies Df(3L)ED4536, Df(3R)ED5559 and Df(3R)ED6076.

| **Df(3L)ED4536** | | **Df(3R)ED5559** | | **Df(3R)ED6076** | |
|---|---|---|---|---|---|
| **Gene Name** | **VDRC stock number** | **Gene Name** | **VDRC stock number** | **Gene Name** | **VDRC stock number** |
| ***CG32141*** | v105447 | ***CG14710*** | N/A | ***Cchl*** | v101382 |
| | v41107 | ***CG14711*** | N/A | | v45020 |
| ***CG8100*** | v101026 | ***CG14712*** | v18347 | | v45021 |
| | v28205 | ***CG18764*** | v107494 | ***CG13409*** | v100569 |
| ***D*** | v107194 | | v33403 | | v8628 |
| | v2940 | ***CG5342*** | v44403 | | v47578 |
| | v49549 | | v50058 | | v47576 |
| ***Fbp1*** | v37881 | | v44404 | | v47577 |
| ***nan*** | v100090 | ***CG6790*** | v107442 | ***CG17819*** | v108496 |
| | v5261 | | v12134 | | v24975 |
| | v5260 | | v12135 | | v45422 |
| ***nuf*** | v104172 | ***CG6791*** | v106627 | | v45424 |
| | v109426 | | v27748 | | v45423 |
| | v28069 | | v45576 | ***CG17843*** | v102838 |
| | v34417 | | v45987 | ***CG31465*** | v21353 |
| ***Sox21a*** | v104888 | ***CG6808*** | v36364 | ***CG34148*** | N/A |
| | v10813 | | v45266 | ***CG45099*** | N/A |
| ***Sox21b*** | v41098 | ***CG6813*** | N/A | ***CG6015*** | v41708 |
| | | ***Jupiter*** | v25044 | ***CG6028*** | v52314 |
| | | | v25045 | | v52313 |
| | | ***Mrp4*** | v101221 | ***CG6332*** | v108472 |
| | | | v6053 | | v43799 |
| | | ***wkd*** | v22081 | ***CG6439*** | v100822 |
| | | | v22082 | | v14443 |
| | | | | ***CG6455*** | v106757 |
| | | | | | v11938 |
| | | | | | v47615 |
| | | | | | v47616 |
| | | | | ***CG6656*** | v104175 |
| | | | | | v1630 |
| | | | | | v1631 |
| | | | | | v1633 |
| | | | | ***CG6678*** | v100804 |
| | | | | | v26719 |
| | | | | ***CG6690*** | v101104 |
| | | | | | v14439 |
| | | | | ***dnd*** | v104311 |
| | | | | ***Eip93F*** | v104390 |
| | | | | | v45857 |
| | | | | | v45858 |
| | | | | | v45855 |

| | | | | | |
|---|---|---|---|---|---|
| | | | | | v45856 |
| | | | | ***Fadd*** | v100333 |
| | | | | | v7926 |
| **Df(3L)ED4536** | | **Df(3R)ED5559** | | **Df(3R)ED6076** | |
| **Gene Name** | **VDRC stock number** | **Gene Name** | **VDRC stock number** | **Gene Name** | **VDRC stock number** |
| | | | | ***fit*** | v14433 |
| | | | | | v14434 |
| | | | | ***how*** | v100775 |
| | | | | ***mRpL35*** | v103388 |
| | | | | | v13102 |
| | | | | | v13443 |
| | | | | ***ND42*** | v14444 |
| | | | | ***P5CDh2*** | N/A |
| | | | | ***pit*** | v106078 |
| | | | | | v27600 |
