# Supplementary material for: A Novel Frizzled-Based Screening Tool Identifies Genetic Modifiers of Planar Cell Polarity in Drosophila Wings
Source: G3 (Bethesda). 2016 Oct 11;6(12):3963–73. doi: 10.1534/g3.116.035535 (PMC5144966; doi:10.1534/g3.116.035535)
Supplement: Supplemental Material [file supp_g3.116.035535_TableS3.pdf]

Table S3: List of genes, VDRC, and Bloomington stock center stocks tested for each gene for DrosDel deficiency Df(3R)ED5177.

**Df(3R)ED5177**	
**Gene Name**	**Stock number**
asl	BL35039
	v25457
Rga	BL35460
	v20826
Atu	v106074
	v17490
CG1427	BL38911
	v105727
	v17456
	v17457
CG2911	v25451
	v25452
kra	v102609
	v25166
	v25165
Spec2	v101359
