# Supplementary material for: A Novel Frizzled-Based Screening Tool Identifies Genetic Modifiers of Planar Cell Polarity in Drosophila Wings
Source: G3 (Bethesda). 2016 Oct 11;6(12):3963–73. doi: 10.1534/g3.116.035535 (PMC5144966; doi:10.1534/g3.116.035535)
Supplement: Supplemental Material [file supp_g3.116.035535_TableS4.pdf]

Table S4: Phenotypes observed in engrailed driven RNAi lines.

Gene Name	Stock number	en-Gal4 29°C	en-Gal4 25°C	en-Gal4 18°C
CG14712	v18347	Lethal (pupa)	Lethal (pupa)	Margen defects and Notches
D dichaete	v107194	Lethal	Lethal	Lethal
	v2940	Lethal	Lethal	Lethal
	v49549	Wild Type (WT)	WT	WT
CG13310	v43959	Only males, no phenotype	Only males, no phenotype	Only males, no phenotype
	v101276	Lethal (pupa)	Lethal (pupa)	Margen defects, Notches and Blisteting
mRpL35	v103388	Lethal (pupa)	Lethal (pupa)	Lethal (pupa)
	v13443	Lethal (pupa)	Lethal (pupa)	Lethal (pupa)
Sem1	v107661	Lethal	Lethal	Lethal
	v31787	Lethal	Lethal	Lethal
	v49153	Lethal	Lethal	Lethal
mRpL12	v100496	Lethal	Lethal	Lethal
	v26684	Lethal	Lethal	Lethal
